# A three-long noncoding RNA signature as a diagnostic biomarker for differentiating between triple-negative and non-triple-negative breast cancers

**DOI:** 10.1097/MD.0000000000006222

**Published:** 2017-03-03

**Authors:** Man Liu, Lu-Qi Xing, Yi-Jing Liu

**Affiliations:** Department of Pathology, The First Affiliated Hospital of Henan University of Science and Technology, Luoyang, Henan, China.

**Keywords:** diagnosis, lncRNAs, receiver operating characteristic analysis, triple-negative breast cancer

## Abstract

**Background:**

Triple-negative breast cancer (TNBC) is an aggressive cancer with unfavorable outcome and it is useful to explore noninvasive biomarkers for its early diagnosis. Here, we identified differentially expressed long noncoding RNAs (lncRNAs) in blood samples of patients with TNBC to assess their diagnostic value.

**Methods:**

Differential expression of lncRNAs in plasma of patients with TNBC (n = 25) and non-TNBC (NTNBC; n = 35) and in healthy controls was compared by microarray analysis and validated by real-time PCR. lncRNA expression between plasma and BC tissues was compared using Pearson correlation test. Logit model was used to obtain a new lncRNA-based score. Receiver operating characteristic analysis was performed to assess the diagnostic value of the selected lncRNAs.

**Results:**

Microarray data showed that 41 lncRNAs were aberrantly expressed. Among these, antisense noncoding RNA in the INK4 locus (*ANRIL*), hypoxia inducible factor 1alpha antisense RNA-2 (*HIF1A-AS2*), and urothelial carcinoma-associated 1 (*UCA1*) were markedly upregulated in plasma of patients with TNBC compared with patients with NTNBC (*P* < 0.01). *HIF1A-AS2* expression was positively associated with its tissue levels (*r* = 0.670, *P* < 0.01). AUC (95% CI) of *ANRIL*, *HIF1A-AS2*, and *UCA1* was 0.785 (0.660–0.881), 0.739 (0.610–0.844), and 0.817 (0.696–0.905), respectively. TNBCSigLnc-3, a new score obtained using the logit model, showed excellent diagnostic performance, with AUC of 0.934 (0.839–0.982), sensitivity of 76.0%, and specificity of 97.1%.

**Conclusion:**

*ANRIL*, *HIF1A-AS2*, and *UCA1* expression was significantly increased in plasma of patients with TNBC, suggesting their use as TNBC-specific diagnostic biomarkers.

## 1 Introduction

Breast cancer (BC) is one of the most common malignant tumors, accounting for 28.6% of all newly diagnosed cancer cases among women in 2015, and is a major cause of cancer deaths among women.^[1]^ Triple-negative BC (TNBC), which lacks estrogen receptor (ER) and progesterone receptor (PR) expression and human epidermal growth factor receptor 2 (Her2) amplification,^[2]^ accounts for approximately 10% to 20% of all BC cases and is characterized by larger tumor size, higher grade, more positive lymph nodes, and poorer prognosis than other BC subtypes.^[3]^ Patients with TNBC do not respond to endocrine or Her2-targeted therapy, and treatment of TNBC involves a combination of commonly used BC therapies, including surgery, radiation, and chemotherapy regimens.^[4]^ Therefore, there is an urgent need to identify novel biomarkers and potential therapeutic targets for treating this aggressive TNBC phenotype.

Accumulating evidence indicates that in addition to short microRNAs, long noncoding RNAs (lncRNAs, which are at least 200-nt long and do not encode proteins but regulate the expression of coding genes) are involved in human tumorigenesis.^[5]^ Functions of lncRNAs mainly include regulation of gene methylation, activation of gene transcription, conjugation with mRNAs and microRNAs to affect translation progression, etc.^[6,7]^ Although prognostic lncRNA expression signatures have been defined for some invasive breast carcinomas,^[5,8]^ limited information is available about lncRNA expression in TNBC.^[9–11]^

The present study determined the feasibility of detecting and quantifying the expression level of lncRNAs in the plasma of patients with BC and assessed 3 lncRNAs (ANRIL, HIF1A-AS2, and UCA1) as novel noninvasive diagnostic biomarkers for differentiating between TNBC and non-TNBC (NTNBC) in the clinical setting. Aberrantly expressed lncRNAs in the plasma of patients with BC were determined by performing microarray analysis and were validated by performing real-time PCR. The identified lncRNAs were investigated as candidate circulating biomarkers for diagnosing TNBC by performing receiver operating characteristic (ROC) and multivariate logistic regression analyses. Our data suggest that lncRNA expression patterns can help identify new molecular biomarkers for diagnosing TNBC.

## 2 Materials and methods

### 2.1 Patients and sample collection

Sixty consecutively hospitalized patients were recruited from The First Affiliated Hospital of Henan University of Science and Technology between July 2014 and December 2015. Inclusion criteria were as follows: female patients with histologically confirmed invasive ductal carcinoma and with an ER-/PR-/Her2- positive phenotype, patients who did not receive any previous treatment, patients without any evidence of metastasis at diagnosis, and patients whose complete clinicopathological data were available. Patients who were previously diagnosed with BC, any other malignant disease, breast carcinoma in situ, or inflammatory BC were excluded. The study also included 40 healthy individuals who served as negative controls. This study was approved by the Research Ethics Committee of the First Affiliated Hospital of Henan University of Science and Technology, and all participants provided written informed consent. Whole peripheral venous blood samples were drawn into gold-top serum-separating tubes. Serum was extracted by centrifugation at 3000 × g for 10 minutes) within 1 hour of blood sample collection and was stored at −80°C for RNA isolation. Specimens obtained during surgery were immediately frozen in liquid nitrogen and were stored at −80°C.

### 2.2 Immunohistochemical analysis

Immunohistochemical analysis was performed to determine ER, PR, and Her2 status by using standard protocols described previously.^[12]^ ER, PR, and Her2 status was confirmed by experienced pathologists. Refer to ER/PR, staining of >5% tumor cell nuclei was considered positive and staining of <5% tumor cell nuclei was considered negative. Her2/Neu staining score of 0 to 2+ was considered negative and of 3+ was considered positive. Nuclear antigen Ki67 values were also obtained, which was widely used in prognosis, predicting of relative responsiveness or resistance to chemotherapy or endocrine therapy, estimating of residual risk in patients on standard therapy.^[13,14]^ Histological/nuclear grading was assessed by performing hematoxylin–eosin staining and by using The Nottingham Grading System.^[15]^ Patients were divided into TNBC (n = 25) and NTNBC (n = 35) groups according to the results of the above analyses.

### 2.3 RNA extraction and complementary DNA synthesis

Total RNA was extracted from BC tissues by using TRIzol reagent (Invitrogen, Carlsbad, CA) or from 400 μL serum samples by using TRIzol LS reagent (Life Technologies, Luoyang), strictly according to the manufacturer's protocol. The purity and quantity of total RNA were estimated by measuring absorbance at 260 (A260) and 280 nm (A280) with NanoDrop 2000 spectrophotometer (Thermo Scientific, Waltham, MA). Complementary DNA (cDNA) was synthesized using 2.5 μg total RNA, TaqMan MicroRNA Reverse Transcription Kit (Applied Biosystems, USA), and random hexamer primers in a final reaction volume of 50 μL. Reverse transcription was performed at 25°C for 10 minutes, 37°C for 120 minutes, and 85°C for 5 minutes, and the cDNA obtained was stored at −20°C.

### 2.4 Microarray analysis

To screen candidate lncRNAs, samples were randomly selected from patients with NTNBC, patients with TNBC, and healthy individuals. Samples with an RNA integrity number of >8 were processed for hybridization. After isolation, lncRNA labeling was performed using Quick Amp Labeling kit (Agilent Technologies, Palo Alto, CA), according to the manufacturer's guidelines, and fluorescence labeling efficiency was determined using NanoDrop spectrophotometer. Hybridization was performed using Human LncRNA Array (v.4.0; Arraystar, Rockville, MD). Scanned images were imported into GenePix 4000B chip scanner (KangChen Bio-tech). Quantile normalization and subsequent data processing were performed using GeneSpringGX v. 11.0 software package (Agilent Technologies) for data analysis.

### 2.5 Real-time PCR

For real-time PCR, 2 μL cDNA solution was mixed with 7.2 μL nuclease-free water, 10 μL iTaq Universal SYBR Green supermix (Bio-Rad, CA), and 0.4 μL forward primer in a final reaction volume of 20 μL, according to the manufacturer's instructions. *GAPDH* was used as an endogenous control for data normalization because its expression level was relatively stable in the plasma. Sequences of primers used are as follows: *ANRIL* forward, 5′-ACACACATCAAAGGAGAATTTT-3′; *ANRIL* reverse, 5′-CCGTCTCTACTGTTACCTC-3′; *HIF1A-AS2* forward, 5′- CTGAGAACTGCTTCACTCA-3′; *HIF1A-AS2* reverse, 5′-TATGTTGTTAGAAAAGAAACATCATT-3′; *UCA1* forward, 5′- GCTTAATCCAGGAGACAAAG-3′; *UCA1* reverse, 5′- CATAGGTGTGAGTGGCG-3′; *GAPDH* forward, 5′- ACTGGCGTCTTCACC-3′; and *GAPDH* reverse, 5′- CGAACATGGGGGCAT-3′. Real-time PCR was performed at 95°C for 2 minutes, followed by 40 cycles of 95°C for 15 seconds and 60°C for 1 minute, in Stratagene Mx3005p Real-Time PCR System (Applied Biosystems, CA). All amplifications were performed in triplicate. Cycle threshold for each lncRNA and *GAPDH* mRNA was recorded, and relative lncRNA expression level was quantified using 2-ΔΔ cycle threshold method.

### 2.6 Statistical analysis

Data were analyzed using SPSS software version 20.0 (SPSS Inc, Chicago, IL). GraphPad Prism 5.0 (GraphPad Software Inc, CA) was used for plotting graphs. Data for continuous variables are presented as mean ± standard deviation. Student *t* test or 2-sided *χ*^2^ test was used to compare differences in plasma lncRNA levels between 2 groups. One-way analysis of variance was used to compare difference among more than 2 groups. Association between lncRNA expression in the plasma and matched BC tissues was determined using Pearson correlation test. ROC curve was constructed, and area under the curve (AUC) was used to assess the diagnostic values of lncRNAs. Multivariate analysis of markers was performed by constructing a logistic regression model, and a new lncRNA score was generated. Regression equation was validated using analysis of variance followed by *t* test. A *P* value of <0.05 was considered statistically significant.

## 3 Results

### 3.1 Clinical characteristics of study subjects

**Figure 1:**
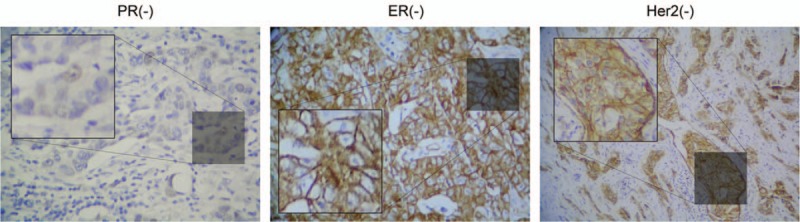
Representative immunostaining patterns in prechemotherapy biopsy samples of patients with triple-negative breast cancer, according to pathological response.

The median age of the patients was 53.4 years (range, 37–76 years). Histopathological diagnosis for all the patients with BC was established by performing core needle biopsy or resection. Results of immunohistochemical analysis showed that 12 patients had luminal A-type BC (ER/PR positivity and Her2 negativity; low score for Ki67), 32 patients had luminal B-type BC (ER/PR positivity and Her2 negativity or positivity; high score for Ki67), and 25 patients had TNBC (ER/PR and Her-2 negativity). Patients with the former 2 types of BC were classified as having NTNBC in the present study. Representative results of immunohistochemical analysis for TNBC are shown in Fig. 1.

### 3.2 LncRNA signature in BC

**Figure 2:**
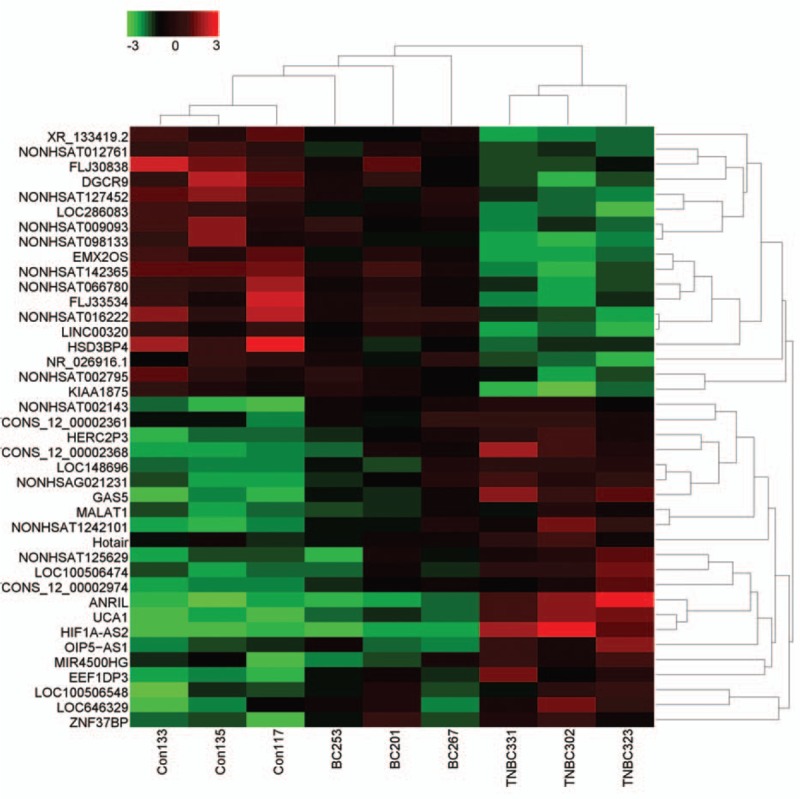
Microarray profiling of lncRNAs in the plasma samples of patients with breast cancer and healthy individuals. A heat map representation of differentially expressed lncRNAs in the 3 study groups; results represent a cutoff *P* value of 0.05 and a fold change of >1.5. Green and red bars indicate downregulated and upregulated lncRNAs, respectively. lncRNAs = long noncoding RNAs.

**Figure 3:**
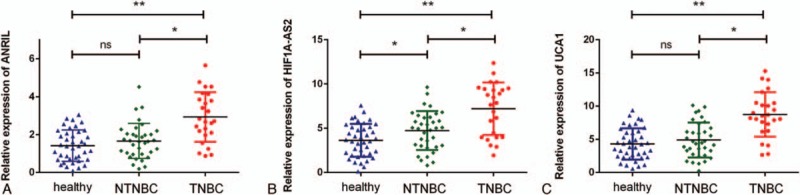
Relative expression levels of *ANRIL*, *HIF1A-AS2*, and *UCA1* in the plasma samples of healthy individuals, patients with triple-negative breast cancer, and patients with non-triple-negative breast cancer. *ANRIL* = antisense noncoding RNA in the INK4 locus, *HIF1A-AS2* = hypoxia inducible factor 1alpha antisense RNA-2, *UCA1* = urothelial carcinoma-associated 1.

**Figure 4:**
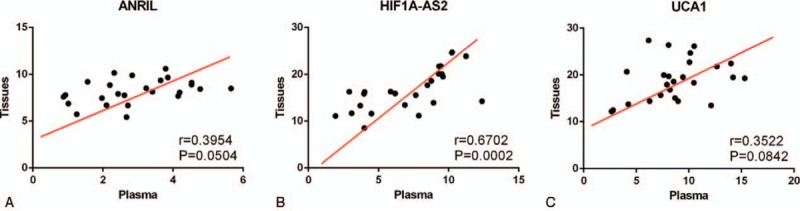
Correlation of lncRNA levels between the plasma and breast cancer tissues of patients with triple-negative breast cancer. lncRNAs = long noncoding RNAs.

To assess the diagnostic value of lncRNAs in BC, 40 healthy individuals were included in the present study. We performed microarray analysis of healthy individuals and patients with NTNBC and with TNBC. Interestingly, microarray data showed that 41 lncRNAs were aberrantly expressed among the 3 study groups. Of these, 19 lncRNAs were downregulated and 22 lncRNAs were upregulated in patients with TNBC compared with those in patients with NTNBC and healthy individuals (fold change, ≥1.5, *P* < 0.05; Fig. 2). Among the upregulated lncRNAs, only 3 lncRNAs, namely, *ANRIL*, *HIF1A-AS2*, and *UCA1*, showed apparent difference in expression between patients with TNBC and NTNBC, thus prompting us to explore their potential for differentiating between these BC subtypes. Expression level of these 3 lncRNAs was determined in plasma samples of 60 patients with BC and 40 healthy individuals by performing real-time PCR. As expected, the relative expression of *ANRIL*, *HIF1A-AS2*, and *UCA1* was significantly higher in patients with TNBC than in patients with NTNBC (*P* < 0.01 for all), which was consistent with the results of microarray analysis. Moreover, statistically significant difference was observed in the relative expression of these lncRNAs between patients with TNBC and healthy individuals. However, the expression level of *HIF1A-AS2* was higher in patients with NTNBC than in healthy individuals (*P* < 0.01; Fig. 3B), which was inconsistent with the results of microarray analysis. The expression level of ANRIL and UCA1 showed no difference between NTNBC patients and healthy individuals (Fig. 3A and C). This discrepancy may be because of false-negative results obtained by performing microarray analysis. Because its ability to differentiate between TNBC and NTNBC, the Spearman correlation test was performed to determine the correlation between the expression levels of these lncRNAs in the plasma and in BC tissues. Results of Pearson correlation test showed that *HIF1A-AS2* expression in the plasma was positively correlated with its tissue levels (*r* = 0.6702, *P* = 0.0002). However, this relationship was not observed for *ANRIL* and *UCA1* (Fig. 4). Furthermore, the expression of these 3 lncRNAs was compared with various clinical parameters. Expression of *HIF1A-AS2* and *UCA1* was higher in patients with lymph node metastasis at the time of diagnosis (*P* < 0.05), and expression of *ANRIL* was higher in patients with BC with high Ki67 score than low one (*P* < 0.05) (Table 1).

**Table 1:**
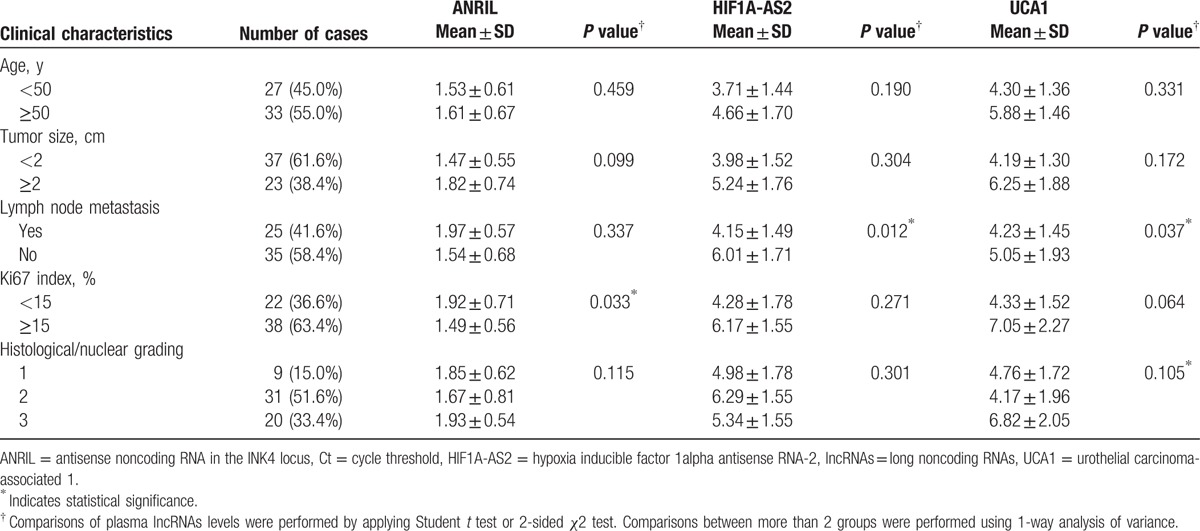
Relation between plasma lncRNAs levels (ΔCt) and clinical characteristics of 60 patients with breast cancer.

### 3.3 Diagnostic performance of lncRNAs in patients with BC

**Figure 5:**
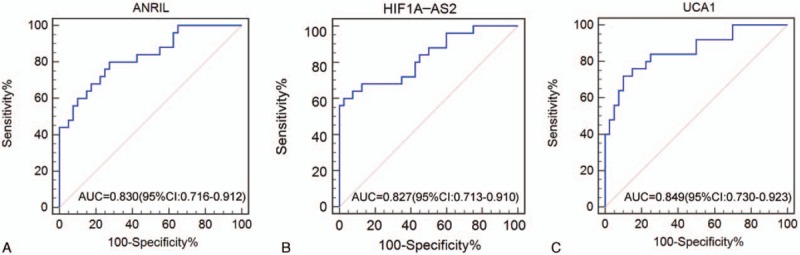
Diagnostic performance of *ANRIL*, *HIF1A-AS2*, and *UCA1* in the plasma samples of healthy individuals and patients with triple-negative breast cancer. Receiver operating characteristic curve analysis. *ANRIL* = antisense noncoding RNA in the INK4 locus, *HIF1A-AS2* = hypoxia inducible factor 1alpha antisense RNA-2, *UCA1* = urothelial carcinoma-associated 1.

**Figure 6:**
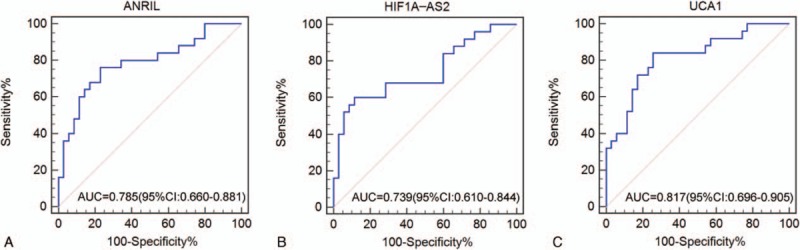
Diagnostic performance of *ANRIL*, *HIF1A-AS2*, and *UCA1* in the plasma samples of patients with triple-negative breast cancer and patients with non-triple-negative breast cancer patients. Receiver operating characteristic curve analysis. *ANRIL* = antisense noncoding RNA in the INK4 locus, *HIF1A-AS2* = hypoxia inducible factor 1alpha antisense RNA-2, *UCA1* = urothelial carcinoma-associated 1.

**Figure 7:**
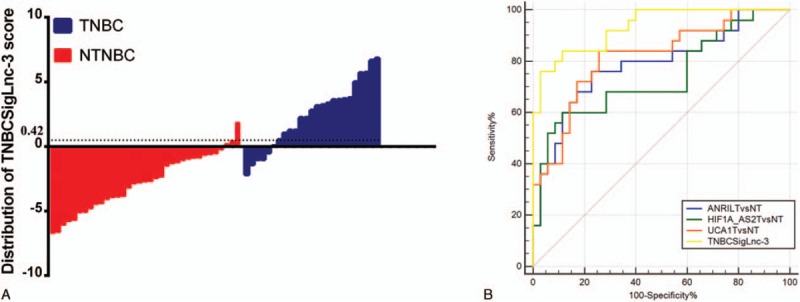
Diagnostic performance of TNBCSigLnc-3 in the plasma samples of patients with breast cancer. A, Orderly distribution of TNBCSigLnc-3 values between patients with triple-negative breast cancer and patients with nontriple-negative breast cancer. B, Pairwise comparison of receiver operating characteristic curves of 4 subjects. TNBC = triple-negative breast cancer.

ROC curves were constructed based on the above findings, and AUC was generated to assess the diagnostic values of the 3 lncRNAs. The AUC values of *ANRIL*, *HIF1A-AS2*, and *UCA1* for differentiating between patients with TNBC and healthy individuals were 0.830 (0.716–0.912), 0.827 (0.713–0.910), and 0.849 (0.730–0.923), respectively (Fig. 5). More attention was paid if these biomarkers could work on distinguishing between TNBC and NTNBC. Therefore, we performed ROC analysis on patients with TNBC and NTNBC and found that the AUC values of *ANRIL*, *HIF1A-AS2*, and *UCA1* were 0.785 (0.660–0.881), 0.739 (0.610–0.844), and 0.817 (0.696–0.905), respectively (Fig. 6). Detailed information on the ability of these 3 lncRNAs to differentiate between patients with TNBC and NTNBC is presented in Table 2. Multivariate logistic regression analysis indicated that the plasma levels of *ANRIL*, *HIF1A-AS2*, and *UCA1* were potential risk factors for TNBC after adjusting for other clinical parameters (Table 3). Although *ANRIL*, *HIF1A-AS2*, and *UCA1* can potentially distinguish between TNBC and NTNBC, their sensitivity and specificity are not high. Therefore, we used the logistic regression model to a new biomarker TNBCSigLnc-3 (−10.25 + 1.07×*ANRIL* + 0.53×*HIF1A-AS2* + 0.65×*UAC1*). Figure 7A shows the orderly distribution of values of TNBCSigLnc-3 among 25 patients with TNBC and 35 patients with NTNBC, with a cutoff value at 0.42. Independent comparison with the 3 lncRNAs showed that TNBCSigLnc-3 had the highest AUC value of 0.934 (0.839–0.982), relatively high sensitivity of 76.0%, and highest specificity of 97.1% (Fig. 7B).

**Table 2:**
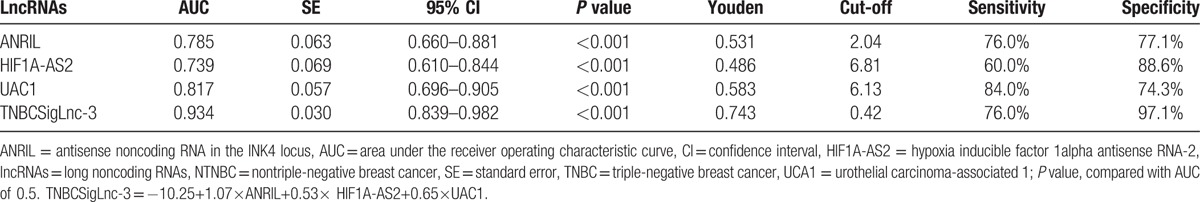
The receiver operating characteristic (ROC) analysis of lncRNA for distinguish TNBC from NTNBC.

**Table 3:**
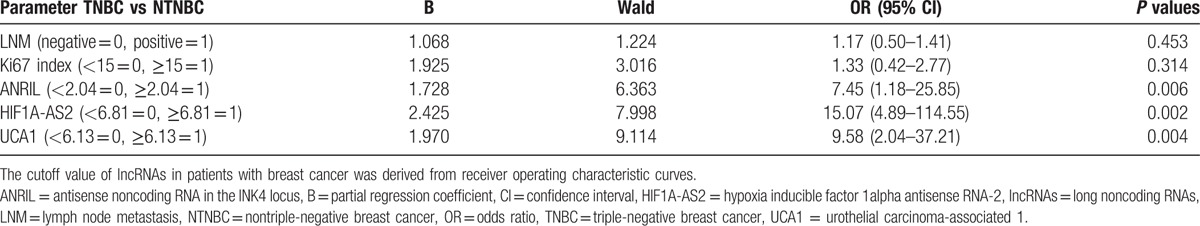
Multivariate logistic analyses for plasma lncRNAs levers in patients with breast cancer.

## 4 Discussion

Accumulating data suggest that deregulation of lncRNAs is associated with the modulation of oncogenic and tumor-suppressing pathways.^[16,17]^ LncRNA signatures of normal cancer tissues and metastases are used to classify different cancer types, indicating the potential of these lncRNAs as biomarkers for diagnosis, prognosis, and therapy.^[18–20]^ One study investigated several lncRNAs expression levels among molecular breast cancer subtypes and indicated that lncRNA LINC00052, RP11-434D9.1, IGKV, and BC016831 could serve as biomarkers for diagnosis for TNBC.^[11]^ Chen et al^[21]^ studied the role of deregulated lncRNAs in TNBC tissues with lncRNA microarray chips and website bioinformatics tools and finally found that lncRNA LINC00993, which was strongly associated with ER expression, played a key role in TNBC. However, few studies focused the expression of lncRNAs associated with TNBC in blood samples.^[8]^ Recent studies have suggested that some lncRNAs are present in serum, plasma, and other bodily fluids in a stable form protected from endogenous RNases, which makes them suitable markers for the noninvasive analysis of patient samples.^[22,23]^ The present study focused on the deregulated expression of lncRNAs in the plasma samples of patients with BC to establish them as novel noninvasive biomarkers for differentiating between TNBC and NTNBC. We performed microarray analysis to determine lncRNA profiles and identified 41 aberrantly expressed lncRNAs. Of these, only *ANRIL*, *HIF1A-AS2*, and *UCA1* were upregulated in patients with TNBC compared with NTNBC, which was validated further by performing real-time PCR. However, only *HIF1A-AS2* expression in the plasma was significantly correlated with its level in BC tissues; moreover, *HIF1A-AS2* showed differential expression in patients with NTNBC and healthy individuals. Nonetheless, research concerning lncRNAs as biomarkers for BC is still in its infancy.

Thus far, *ANRIL* upregulation is considered the primary feature of many carcinomas, including BC.^[24]^ Lin et al^[25]^ found that *ANRIL* expression was higher in nonsmall cell lung cancer tissues than adjacent nontumor tissues and was associated with high TNM stage and advanced lymph node metastasis. Similarly, *ANRIL* expression was higher in hepatocellular carcinoma tissues than in adjacent tumor-free tissues, and patients with high *ANRIL* expression showed significantly poor overall survival.^[26]^ In addition, *ANRIL* overexpression in patients with serous ovarian cancer was associated with an aggressive tumor phenotype and poor prognosis. An in vitro study suggested that *ANRIL* plays an important role in regulating cell migration/invasion by regulating Epithelial-Mesenchymal Transition (MET) and Matrix Metalloproteinase-3 in serous ovarian cancer.^[27]^ Studies have also shown that *ANRIL* knockdown significantly inhibits the proliferation, invasion, and metastasis of gastric cancer cells^[28]^ and thyroid cancer cells.^[29]^ Importantly, Royds et al^[30]^ investigated rs11515 single nucleotide polymorphism in breast tumors and suggested that this polymorphism was more frequent and was associated with an aggressive tumor phenotype because of increased *ANRIL* and decreased *p16*^*INK4a*^ expression. A recent study measured mRNA levels of the gene encoding *ANRIL* in 456 breast carcinomas tissues and found that *ANRIL* mRNA expression was higher in breast carcinomas tissues than in normal breast tissues, which was exclusively and weakly correlated with ER and PR status and showed a complex association with epithelial–mesenchymal transition markers.^[31]^ We determined *ANRIL* expression in the plasma of patients with BC and found that its expression was surprisingly higher in patients with TNBC than in patients with NTNBC. These findings prompted us to determine the diagnostic value of *ANRIL* in TNBC, which has not been performed to date. We also obtained similar results for *HIF1A-AS* and *UCA1*. The antisense long noncoding RNA hypoxia inducible factor 1alpha antisense RNA-2 (*HIF1A-AS2*), which was located in chromosome 14q23.2, was reported overexpressed in several tumor tissues, such as chronic myeloid leukemia and neuroblastoma,^[32]^ but the studies about the exact significance of *HIF1A-AS2* ware limited. Antisense lncRNA *HIF1A-AS2* is highly expressed in gastric cancer, and its expression is correlated with TNM stage, tumor invasion, lymph node metastasis, and poor prognosis and knockdown of HIF1A-AS2 expression by siRNA could inhibit cell proliferation in vitro and tumorigenesis in vivo.^[33]^ In addition, a recent study determined the tumor suppressor function of *HIF1A-AS2* in glioblastoma multiforme^[34,35]^ and similarly, researchers suggested that silencing *HIF1A-AS2* could lead to cell proliferation inhibition, cell migration suppression, and apoptosis induction in bladder cancer cells.^[35]^ To our knowledge, no study has explored the function of *HIF1A-AS2* in BC to date. The present study is the first to investigate *HIF1A-AS2* expression in patients with TNBC and NTNBC. ROC analysis generated a relatively satisfied diagnostic value of *HIF1A-AS2* for TNBC. Urothelial carcinoma-associated 1 (*UCA1*) is a new lncRNA-encoding gene belonging to human endogenous Retrovirus-H family and was originally identified in bladder transitional cell carcinoma. Because *UCA1* is highly expressed in bladder transitional cell carcinoma, it was suggested as a biomarker for diagnosing bladder cancer.^[36]^ Zheng et al^[37]^ detected *UCA1* expression in 112 pairs of tumorous and adjacent normal tissues of patients with gastric cancer and found that high *UCA1* expression was correlated with poor differentiation, tumor size, invasion depth, TNM stage, and poor overall survival. Similarly, *UCA1* expression was upregulated in BC tissues, indicating that *UCA1* plays an oncogenic role in BC both in vitro and in vivo.^[38]^ One study found that *UCA1* promoted the invasiveness of BC cells.^[39]^ Another study showed that endogenous *UCA1* knockdown significantly reduced the number of invading cells, suggesting that *UCA1* upregulation increased the invasiveness of BC cells by activating Wnt/β-catenin signaling pathway.^[40]^ Li et al^[41]^ investigated the expression level of *UCA1* in acquired tamoxifen resistance in estrogen receptor (ER)-positive breast cancer cells and argued that downregulation of *UCA1* could enhance the sensitivity of breast cancer cells to tamoxifen resistance directly interact with miR-143. In contrast, Lee et al^[42]^ suggest that special AT-rich sequence binding protein 1 is the upstream regulator of *UCA1* expression and depletion of *UCA1* could suppress tumor growth and cell survival of breast cancer cells. Consistently, we found that *UCA1* expression was upregulated in the plasma of patients with TNBC compared with that in healthy individuals. Based on this finding, we performed ROC analysis to assess the diagnostic values of the 3 lncRNAs. We found that the 3 lncRNAs had good diagnostic ability to differentiate between patients with TNBC and healthy individuals. Moreover, we explored the diagnostic value of these lncRNAs for distinguishing between TNBC and NTNBC. ROC analysis showed that the AUC values of *ANRIL*, *HIF1A-AS2*, and *UCA1* were 0.785 (0.660–0.881), 0.739 (0.610–0.844), and 0.817 (0.696–0.905), respectively. Moreover, multivariate logistic regression analysis indicated that plasma levels of *ANRIL*, *HIF1A-AS2*, and *UCA1* were risk factors for TNBC after adjusting for other parameters (Table 2). Next, we constructed a regression equation (TNBCSigLnc-3 = −10.25 + 1.07×*ANRIL* + 0.53×*HIF1A-AS2* + 0.65×*UAC1*) based on these 3 lncRNAs. The AUC value of TNBCSigLnc-3 was 0.934 (0.839–0.982), which was superior to that of *ANRIL*, *HIF1A-AS2*, and *UCA1* alone. To our knowledge, our study is the first to show that plasma lncRNAs *ANRIL*, *HIF1A-AS2*, and *UCA1* have excellent diagnostic value for TNBC.

However, this study has a limitation. On one hand, as a pilot study, we explored the diagnostic value of lncRNAs in a small cohort and did not confirm our findings in another group with a larger number of subjects. On the other hand, as time limited, we did not systematically evaluate their predictive value for prognosis or response to chemotherapy in patients with TNBC, which would have provided useful alternatives for personalized treatment of this heterogeneous malignancy. We will focus on these aspects in our future studies.

## 5 Conclusion

In summary, our results expand the findings of previous studies regarding the role of lncRNAs in BC, especially TNBC, and show that the expression of circulating lncRNAs is deregulated in BC. Our data indicate the ideal diagnostic value of lncRNAs *ANRIL*, *HIF1A-AS2*, and *UCA1* to differentiate between patients with TNBC and NTNBC. Because of the high diagnostic value of combined lncRNA analyses in the present study, we anticipate that lncRNA analyses will have great potential in characterizing circulating markers for TNBC.
